# Supplementary material for: Petal size is controlled by the MYB73/TPL/HDA19-miR159-CKX6 module regulating cytokinin catabolism in Rosa hybrida
Source: Nat Commun. 2023 Nov 4;14:7106. doi: 10.1038/s41467-023-42914-y (PMC10625627; doi:10.1038/s41467-023-42914-y)
Supplement: Supplementary file 1 — Supplementary Information [file 41467_2023_42914_MOESM1_ESM.pdf]

# Petal size is controlled by the MYB73/TPL/HDA19-miR159-CKX6 module regulating cytokinin catabolism in *Rosa hybrida*

## Contents

**Supplementary Figures and Legends**

**Uncropped Scans of All Blots and Gels in Supplementary Figures**

# Supplementary Figures and Legends

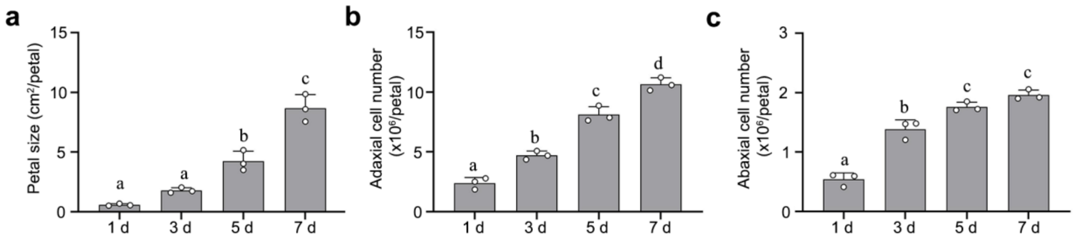


**Supplementary Figure 1 Dynamic of petal size and cell number of adaxial and abaxial epidermal cells during petal growth.**

**a**, Petal size of rose during flower development.

**b**, **c**, Cell numbers of adaxial epidermis (b) and abaxial epidermis (c) during rose petal development.

Different development stages of rose petals are days 1, 3, and 5 after floral bud reached stage 0. Data are shown as means ± SD (n = 3).

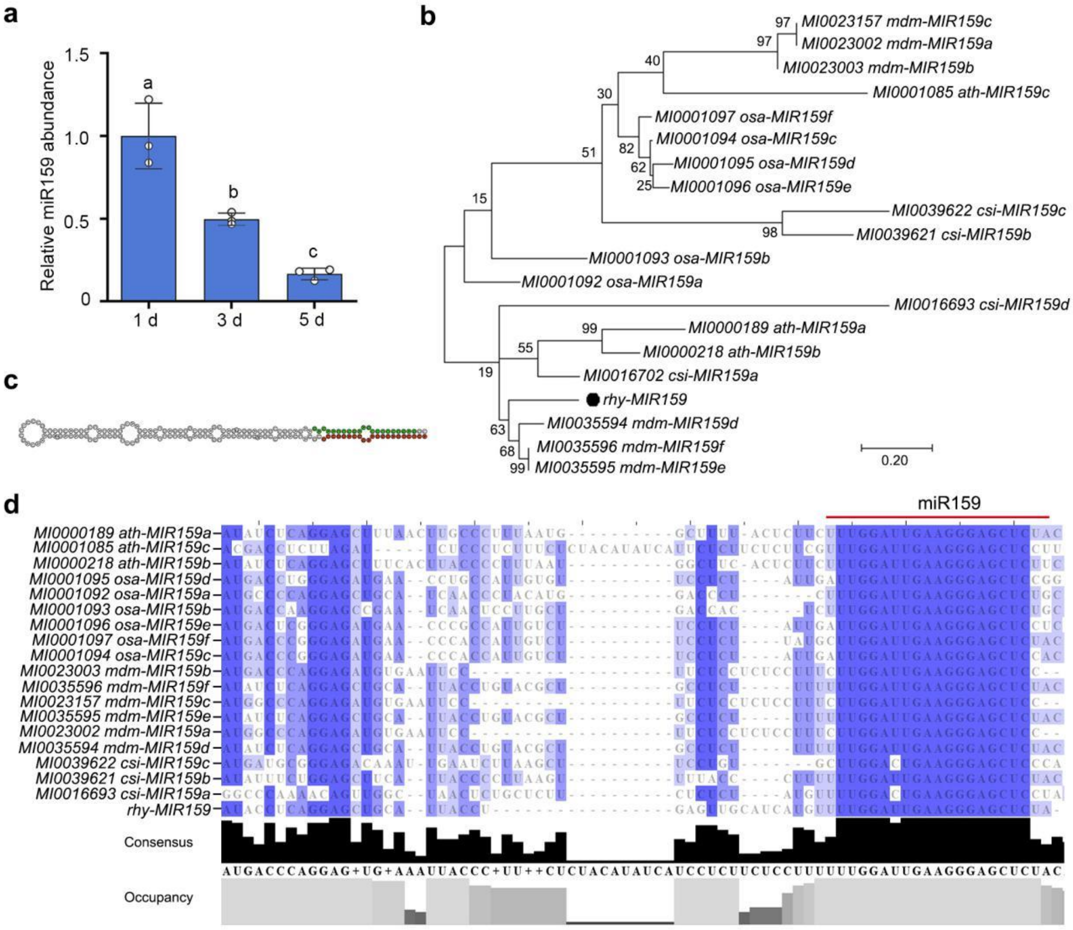


**Supplementary Figure 2 Characterization of *MIR159* in rose.**

**a**, Quantitative RT-PCR (RT-qPCR) analysis of miR159 levels in rose petals on 1, 3, 5 days after floral bud reached stage 0. Data are shown as means ± SD (n = 3). *5S rRNA* was used as an internal control. Different letters above each bar indicate significant differences according to one-way ANOVA with Tukey's multiple comparisons test ($P < 0.05$).

**b**, Phylogenetic analysis of *rhy-MIR159* with other precursors of *MIR159*. The phylogenetic tree was constructed using MEGA software version X with the maximum likelihood algorithm. Bootstrap values indicate the confidence of each branch, and the scale indicates the branch length.

**c**, The predicted secondary structure of *rhy-MIR159*.

**d**, Alignment analysis of miR159 precursor sequence. Red line indicates the sequence of mature miR159.

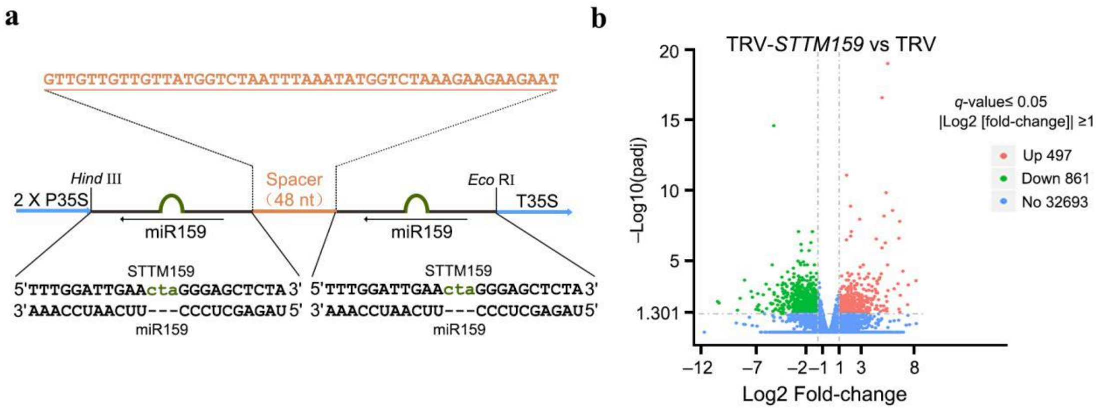


**Supplementary Figure 3 Silencing of *MIR159* in rose petals.**

**a**, Schematic representation of STTM (Short tandem target mimic) structure of miR159 inserted into the TRV vector.

**b**, Up- and down- regulated DEG number in comparison analysis between TRV-*STTM159* and TRV petals, whose samples with three biological replicates (n = 3) were collected at 3 d after stage 0 for RNA sequencing.

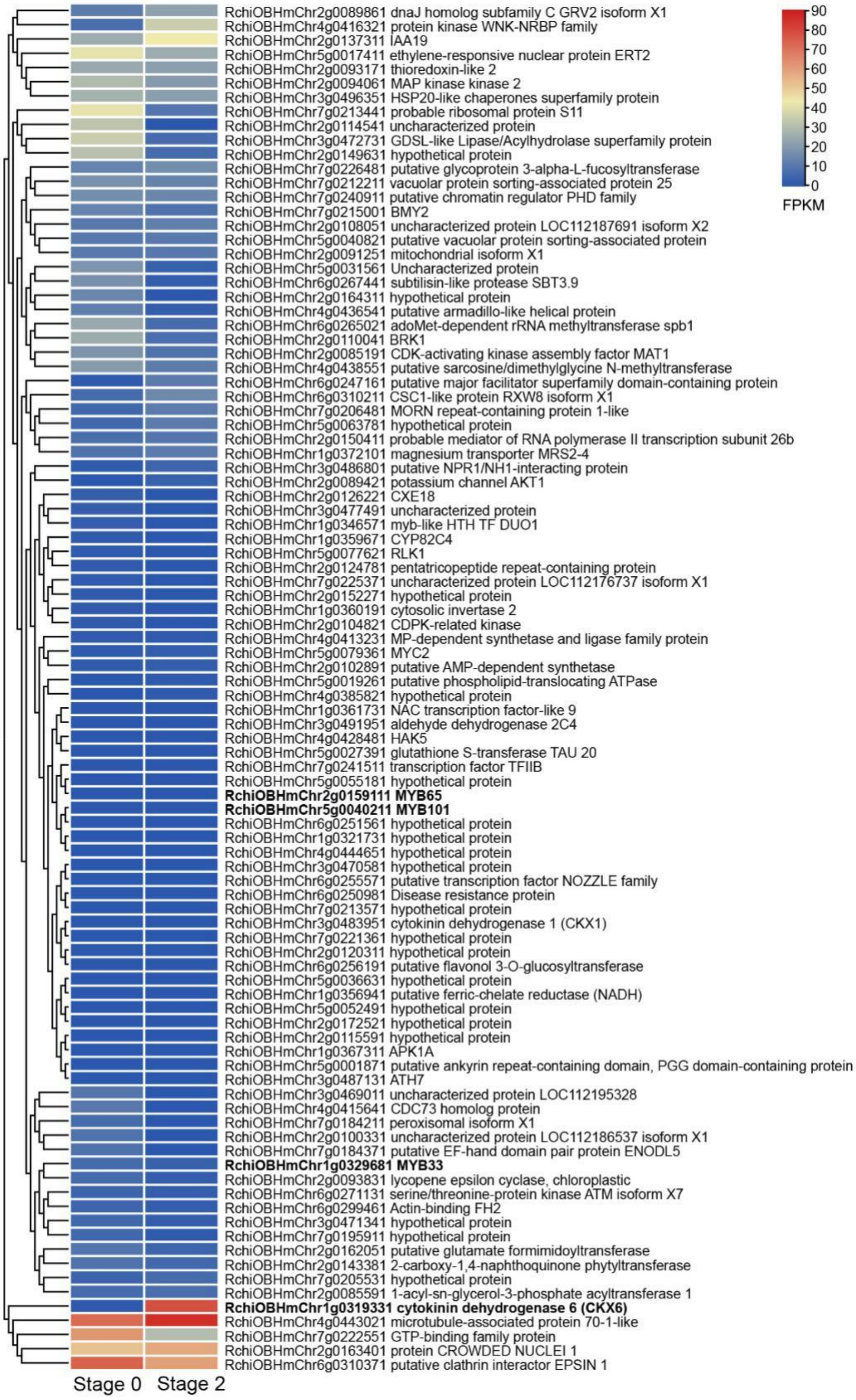


**Supplementary Figure 4 The expression profiles of predicted miR159 targets in petals at stage 0 and stage 2.**

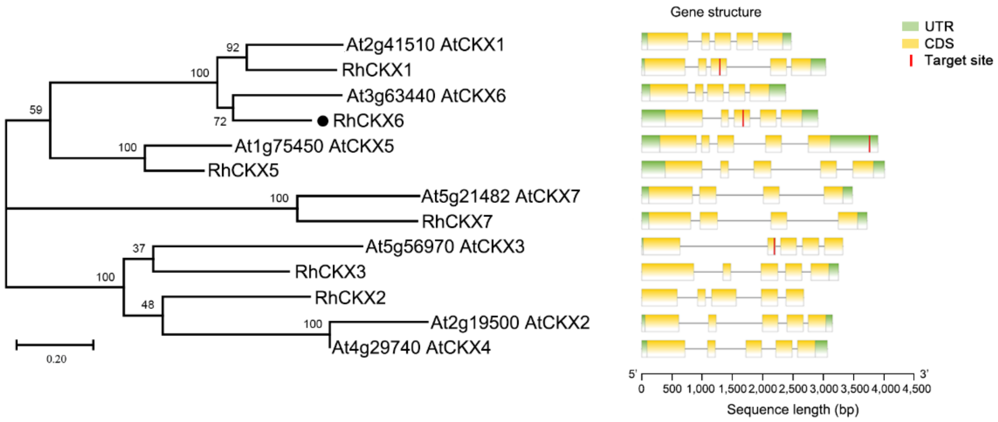


**Supplementary Figure 5 Prediction of miR159 target site in *CKX* transcripts.**

Phylogenetic analysis of CKX proteins in rose and Arabidopsis (Left) using MEGA software version X with the maximum likelihood algorithm. Bootstrap values indicate the confidence of each branch, and the scale indicates branch length. Gene structure (Right) shows the predicted miR159 target site in *CKX* transcripts by using psRNATarget online tool (https://www.zhaolab.org/ psRNATarget/) and visualization analysis (TBtools software version 1.098). Scale bar below the gene structures represents the gene length.

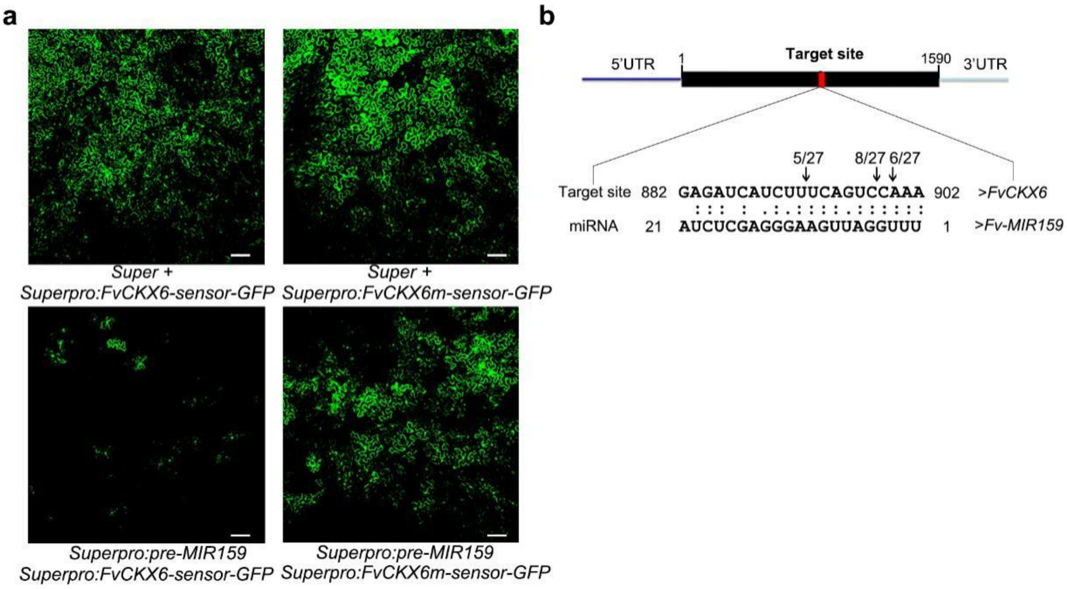


**Supplementary Figure 6 miR159 targets *FvCKX6 in planta*.**

**a,** Confocal imaging analysis of *N. benthamiana* leaves 3 days after co-infiltration of *pre-MIR159* with *FvCKX6-sensor-GFP* or *FvCKX6m-sensor-GFP*.

**b,** Validation of miR159-targeted cleavage of *FvCKX6*. Upper panel, schematic diagram of *FvCKX6* mRNA. Red box, predicted cleavage site of miR159. Bottom panel, identification of cleavage sites using 5′ RLM-RACE assay in strawberry petals. The positions of cleavage sites are indicated by arrowheads with the frequency of clones.

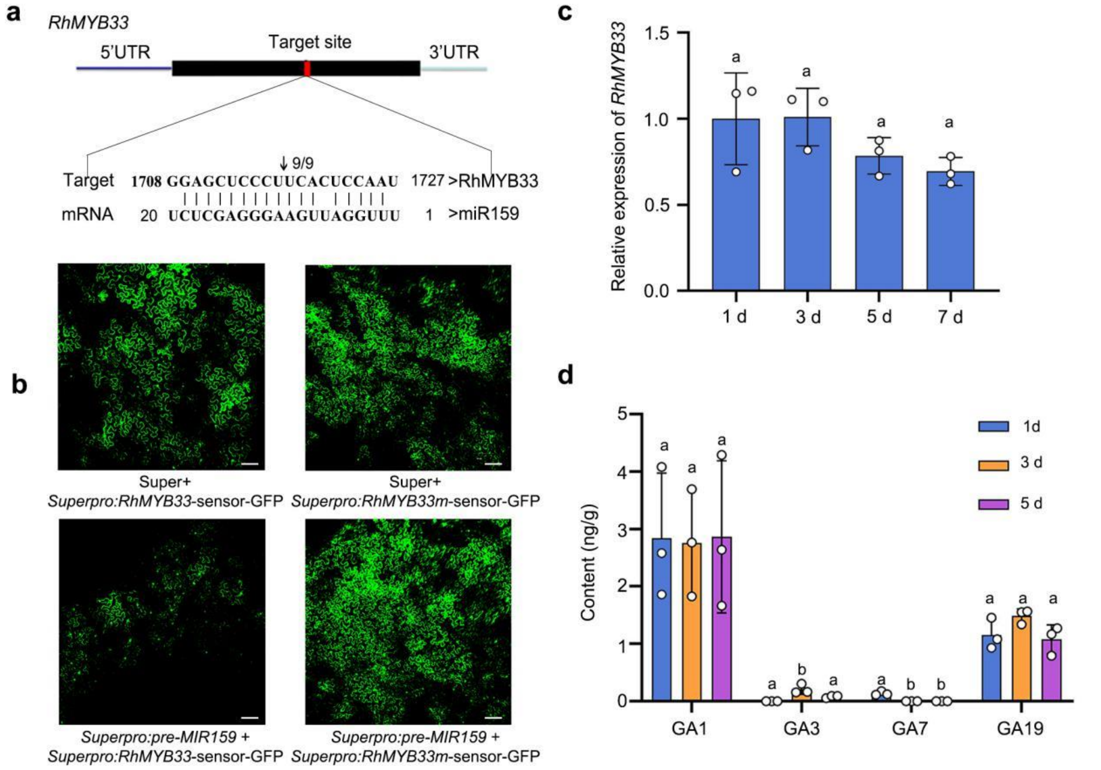


**Supplementary Figure 7 miR159-targeted *RhMYB33* and GAs contents in rose petals during petal growth.**

**a,** Upper panel, schematic diagram of *RhMYB33* mRNA. Red box, predicted cleavage site of miR159. Bottom panel, identification of cleavage sites using 5′ RLM-RACE assay in rose petals. The positions of cleavage sites are indicated by arrowheads with the frequency of clones.

**b,** Confocal imaging analysis of *N. benthamiana* leaves 3 days after co-infiltration of *pre-MIR159* with *RhMYB33-sensor-GFP* or *RhMYB33m-sensor-GFP*, respectively. GFP fluorescence of *N. benthamiana* leaves 3 days after co-infiltration of the indicated constructs. The experiment was performed independently three times, and representative results are shown. Scale bars, 5 mm.

**c,** RT-qPCR analysis of *RhMYB33* transcript levels in petals 1, 3, 5 days after stage 0. *RhUBI2* was used as an internal control. Data are shown as means ± SD (n = 3). Different lowercase letters above each bar indicate significant differences according to one-way ANOVA with Tukey's multiple comparisons test ($P < 0.05$).

**d,** GA contents in petals 1, 3, 5 days after stage 0. Data are shown as means ± SD (n = 3).

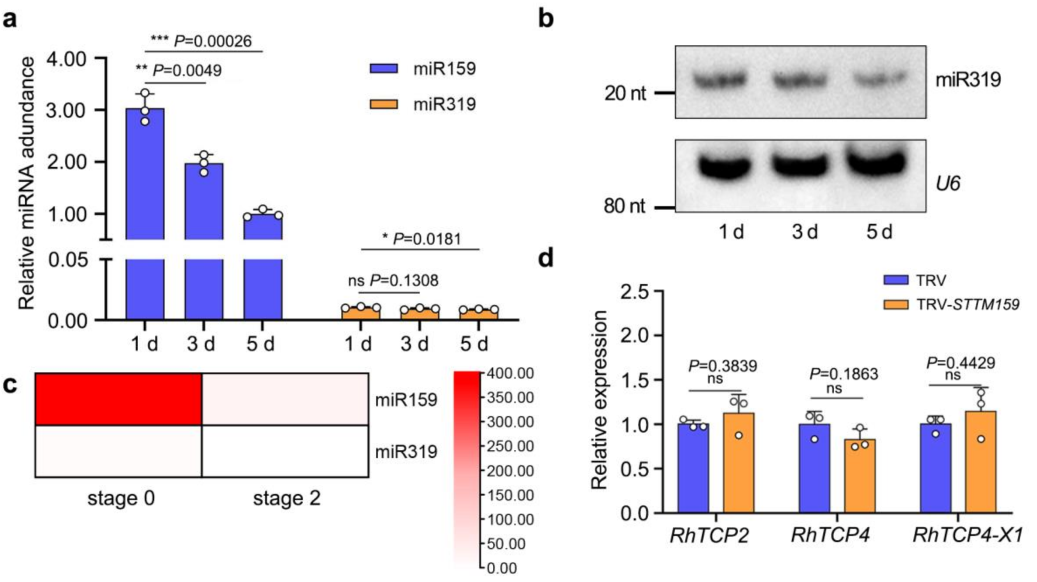


**Supplementary Figure 8 Northern Blot of miR319 and expression of *TCP* genes in petals of miR159-silenced lines.**

**a,** RT-qPCR analysis of miR159 and miR319 levels in rose petals (1, 3 and 5 days after stage 0). Data are shown as means ± SD (n=3). *5S rRNA* was used as an internal control. Asterisks indicate statistically significant differences (two-sided Student's *t*-test, *, $P < 0.05$; **, $P < 0.01$; ***, $P < 0.001$; ns, no significant difference).

**b,** Northern blotting analysis of miR319 abundance in rose petals on 1, 3, and 5 day after floral bud reached stage 0. *U6* was used as control. Locked Nucleic Acids (LNA)-modified oligonucleotide probes were used to ensure the specificity of miR319.

**c,** Heatmap analysis of miR159 and miR319 abundance in rose stage 0 and stage 2.

**d,** RT-qPCR analysis of *RhTCPs* in TRV-*STTM159*. *RhUBI2* was used as an internal control. Data are shown as means ± SD (n = 3).

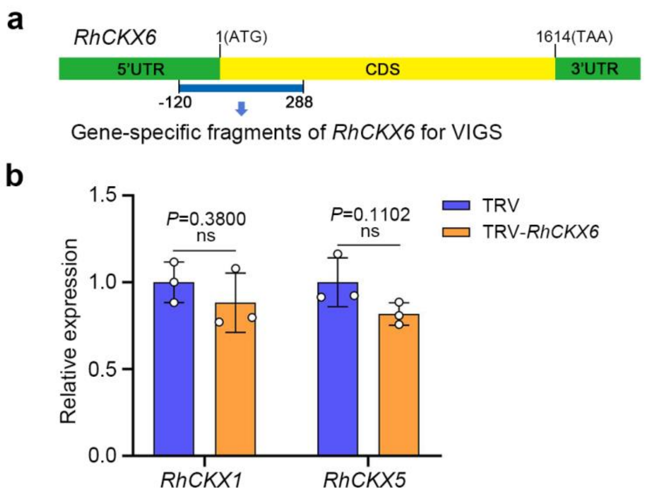


**Supplementary Figure 9 Expression of *RhCKX1* and *RhCKX5* in TRV and *RhCKX6*-silenced lines.**

**a,** Schematic representation of the gene-specific fragment of *RhCKX6* for construction of the TRV-*CKX6* vector.

**b,** Expression of *RhCKX1* and *RhCKX5* in TRV and *RhCKX6*-silenced plants. *RhUBI2* was used as an internal control. Data are shown as means ± SD (n = 3) (two-sided Student's *t*-test, ns, no significant difference).

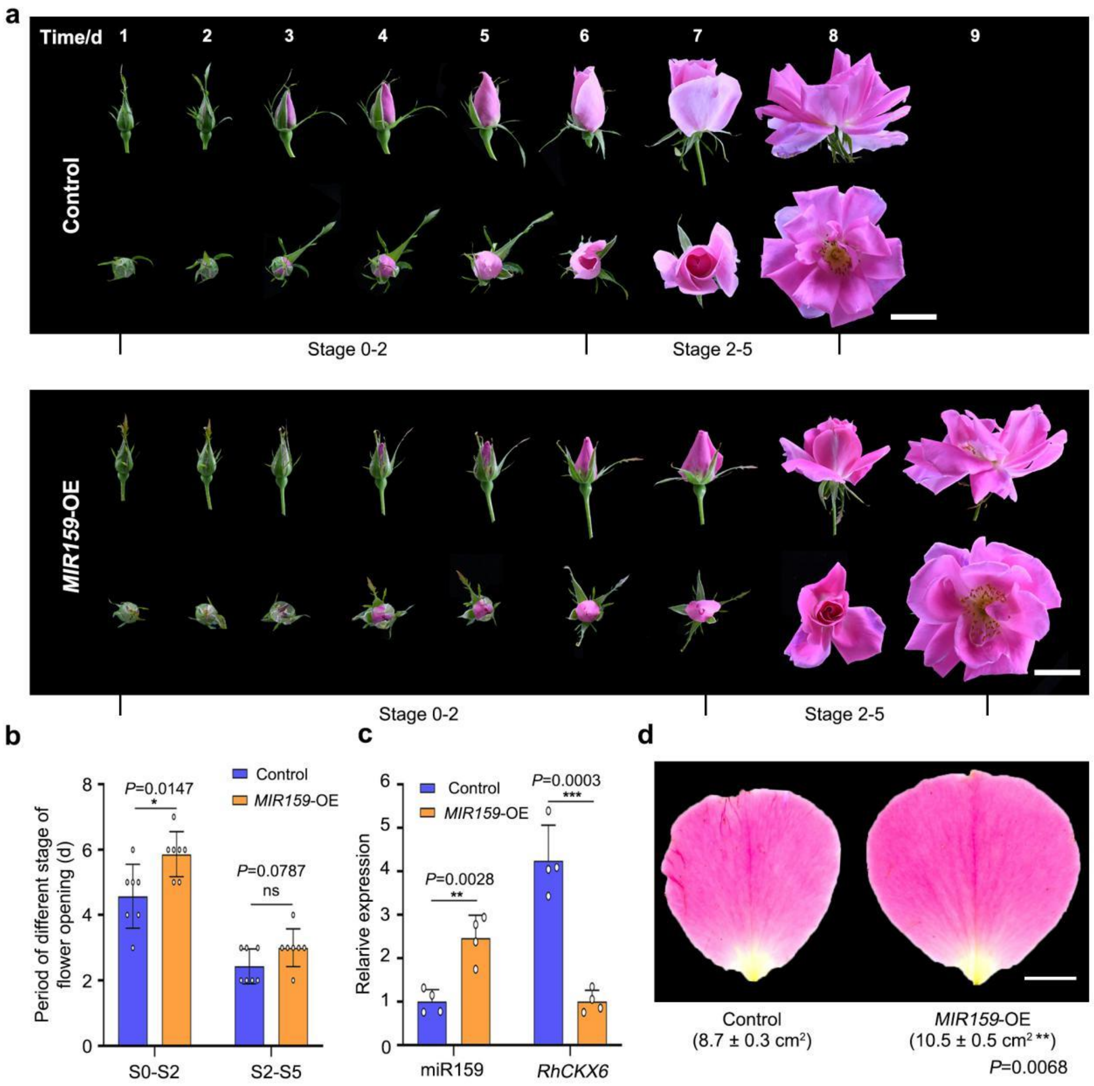


**Supplementary Figure 10 Overexpression of *MIR159* in rose petals.**

**a, b,** Flower opening progression of Control and *MIR159*-OE plants. The experiments were performed independently twice with similar results, and one representative set of results is shown. Scale bars, 2 cm. Data are shown as means ± SD (n = 7) in **b**.

**c,** RT-qPCR analysis of miR159 abundance and *RhCKX6* transcript levels in petals of Control and *MIR159*-OE plants. *5S rRNA* was used as an internal control of miR159. *RhUBI2* was used as an internal control of *RhCKX6*. Data are shown as means ± SD (n = 3).

**d,** Petal size of Control and *MIR159*-OE plants at the fully opened stage. Data are shown as means ± SD (n = 3). The numbers below the images indicate the petal size. Scale bar, 1 cm. (two-sided Student's *t*-test, *, $P < 0.05$; ** $P < 0.01$**).**

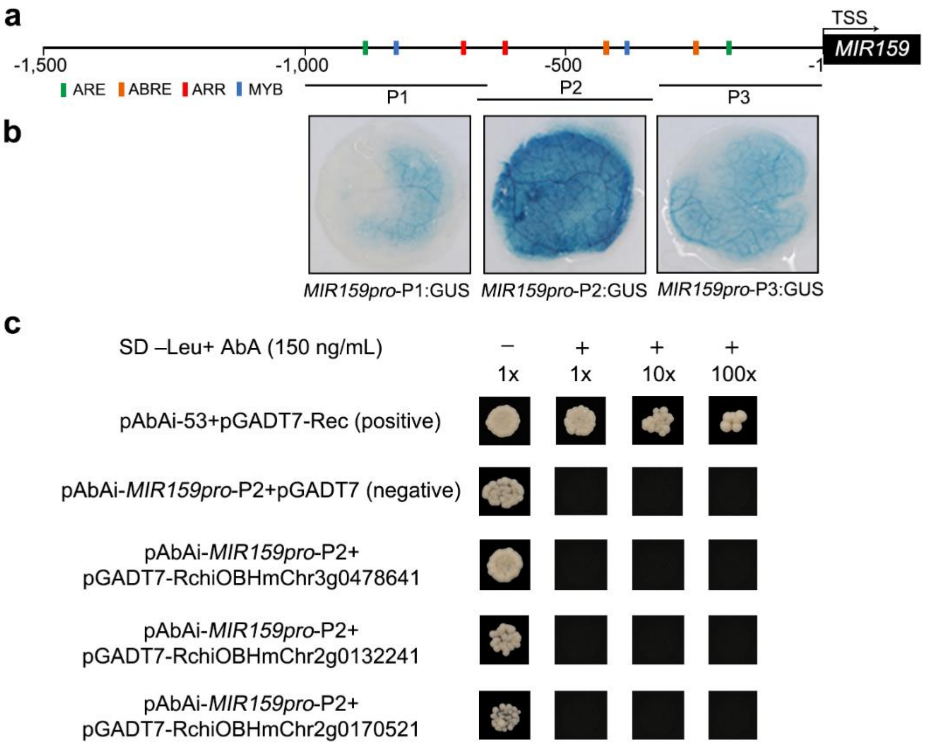


**Supplementary Figure 11 Transactivation analysis and up-stream regulators verification of *MIR159*.**

**a**, Predicted *cis*-elements in the promoter of *MIR159*.

**b**, Transactivation analysis of truncated promoter fragments of *MIR159*.

*MIR159* promoter (–1 to –1000 bp) was truncated into three short segments (P1, P2, P3), and were constructed into *35Smini*:*GUS* vector and were infiltrated into rose petals. GUS staining was taken 3 days after infiltration of rose petals.

**c,** Y1H analysis between candidate proteins (RchiOBHmChr3g0478641, RchiOBHmChr2g0132241, and RchiOBHmChr2g0170521) with the *MIR159* promoter. “+” and “-” indicates SD medium lacking Leu with or without 150 ng $mL^{-1}$ Aureobasidin A (AbA). 1x, 10x, and 100x indicates that the yeast have been diluted 1, 10, and 100 times.

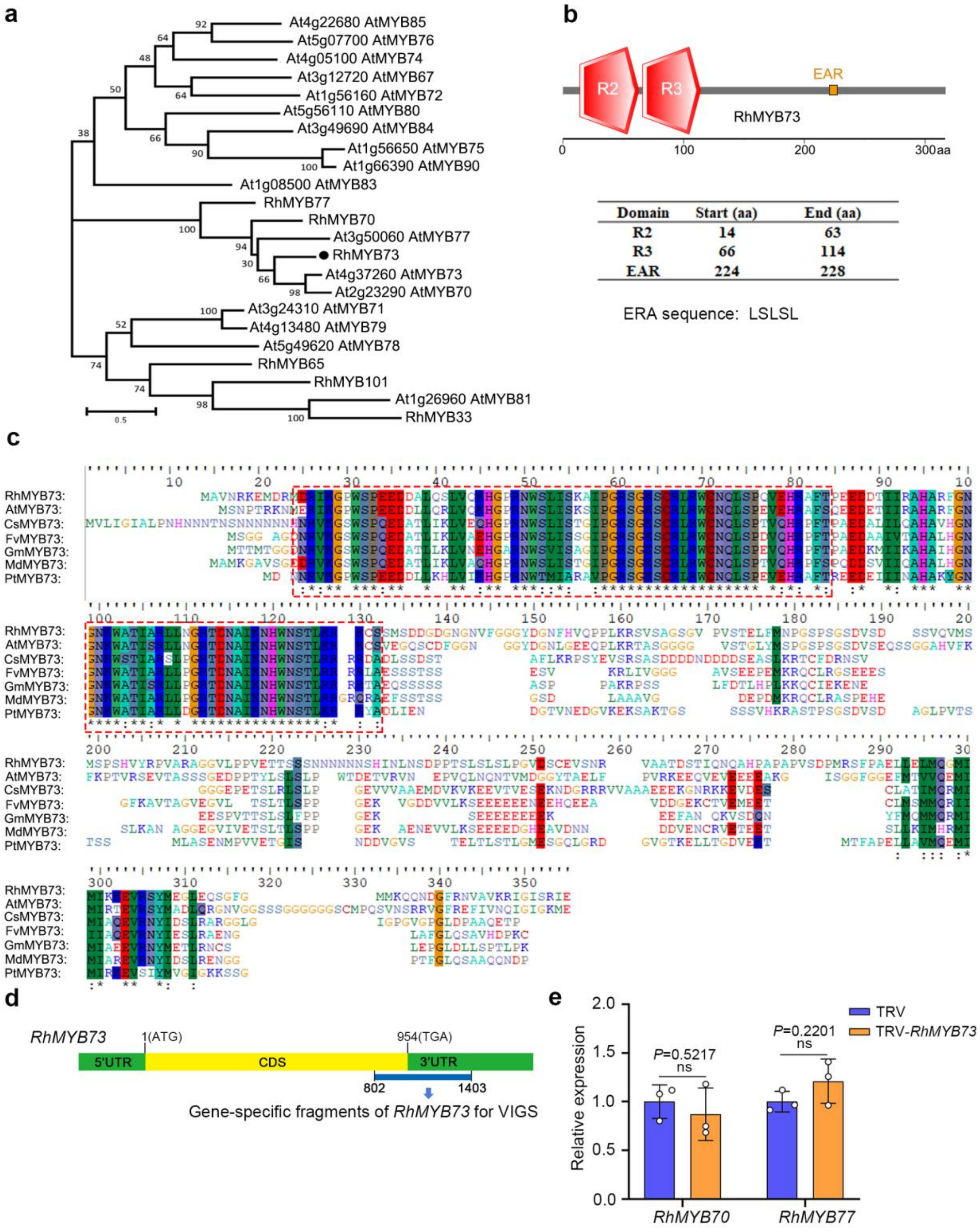


**Supplementary Figure 12 Characterization of RhMYB73.**

**a**, Phylogenetic analysis of RhMYB73. Bootstrap values indicate the confidence of each branch, and the scale indicates branch length. Black dot represents the RhMYB73.

**b**, Conserved domain analysis of RhMYB73 protein, including location of different domains (upper and middle) by using website prediction (http://smart.embl-heidelberg.de/). Scale bar represents the protein length. The bottom shows the EAR sequence: LSLSL.

**c**, Alignment of putative amino acid sequences of RhMYB73 protein with other 6 species: AtMYB73 (AT4G37260.1), *A. thaliana*; CsMYB73 (XP_004148201.1), *Cucumis sativus*;

FvMYB73 (XM_011472328.1), *Fragaria vesca*; GmMYB73 (XP_006595859.1), *Glycine max*; MdMYB73 (XP_008374172.2), *Malus domestica*; PtMYB73 (XP_002313145.1), *Populus trichocarpa*. The conserved MYB domain was showed using dashed box, and the conserved degree of amino acid sites was aligned using BioEdit sequence alignment editor software. Amino acids that are similar in 100% of aligned sequences with same color background.

**d,** Schematic representation of the gene-specific fragment of *RhMYB73* for construction of the TRV-*MYB73* vector.

**e,** Expression of *RhMYB70* and *RhMYB77* in *RhMYB73*-silenced plants. *RhUBI2* was used as an internal control. Data are shown as means ± SD (n = 3) (two-sided Student's *t*-test, ns, no significant difference).

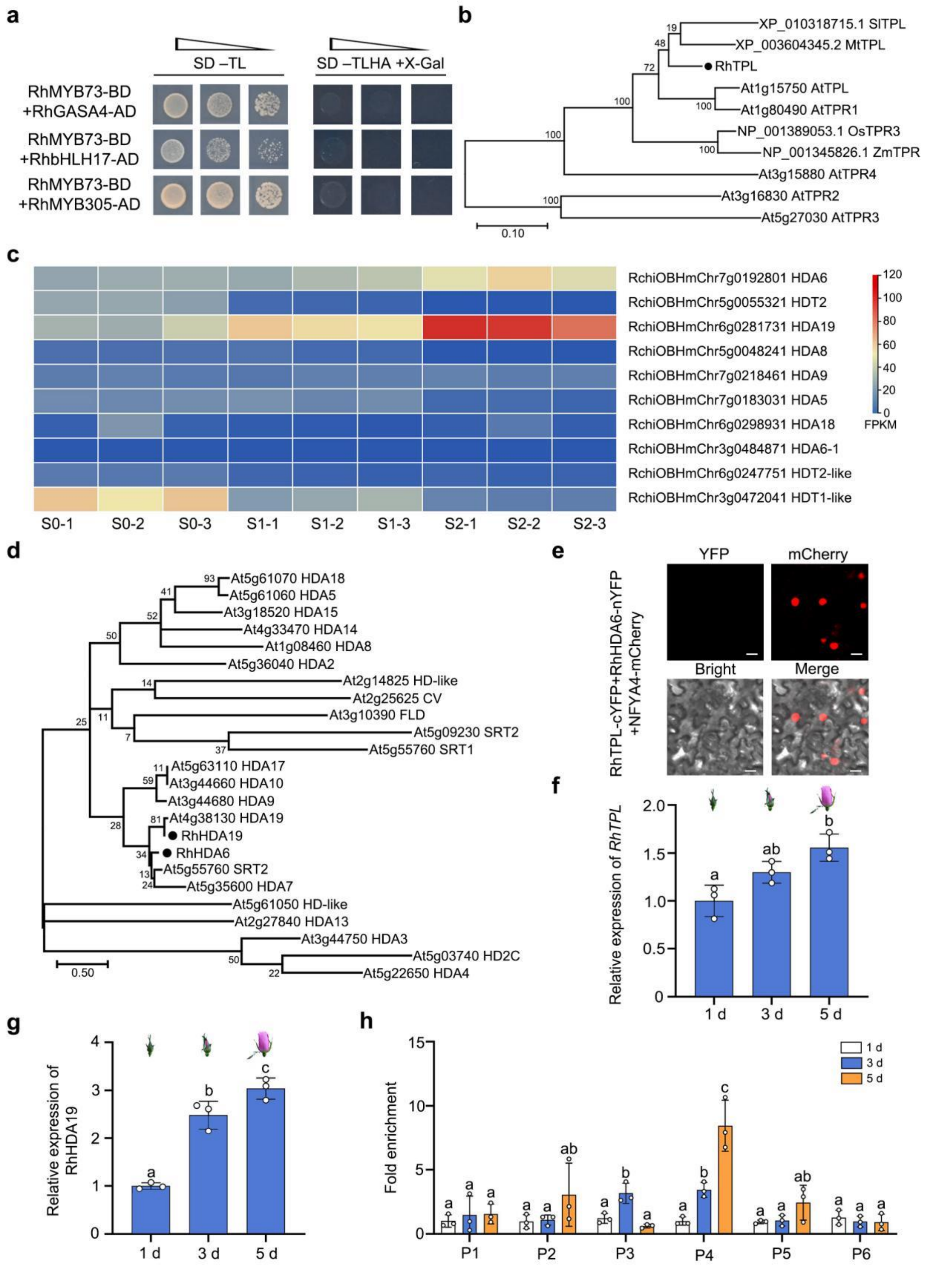


**Supplementary Figure 13 Characterization of *RhHDA19* and *RhTPL*.**

**a**, Y2H assay for the possible interaction between RhMYB73 and candidate proteins from IP-MS.

**b**, Phylogenetic tree analysis of RhTPL. Bootstrap values indicate the confidence of each branch, and the scale indicates branch length.

**c**, Expression profiles of predicted *HDAC* genes in petals during early development phase.

**d**, Phylogenetic tree analysis of RhHDA19. Bootstrap values indicate the confidence of each branch, and the scale indicates branch length.

**e**, Bimolecular fluorescence complementation assay of interaction between RhTPL and RhHDA6. *N. benthamiana* leaves were co-infiltrated with RhTPL-cYFP and RhHDA6-nYFP constructs. The visual imaging was observed by confocal microscopy 3 d after infiltration. Scale, 20 μm. The experiment was performed independently three times, and representative results are shown.

**f**, **g**, Quantitative RT-PCR analysis of *RhTPL* (**f**) and *RhHDA19* (**g**) in rose petals during early development phase (1 d, 3 d, 5 d after Stage 0). *RhUBI2* was used as an internal control. The mean values ± SD are shown from three biological replicates (n = 3). Different letters above each bar in (**f** and **g**) indicate significant differences according to one-way ANOVA with Tukey's multiple comparisons test ($P < 0.05$).

**h**, ChIP-quantitative PCR assay of the relative H3K14ac level in the *MIR159* promoter in rose petals during early development phase (1 d, 3 d, 5 d after Stage 0). The experiment was performed independently twice with similar results, and one representative result is shown. The mean values ± SD are shown from three biological replicates (n = 3). Different lowercase letters above bars of each region (P1 to P6) indicate significant differences according to one-way ANOVA with Tukey's multiple comparisons test ($P < 0.05$).

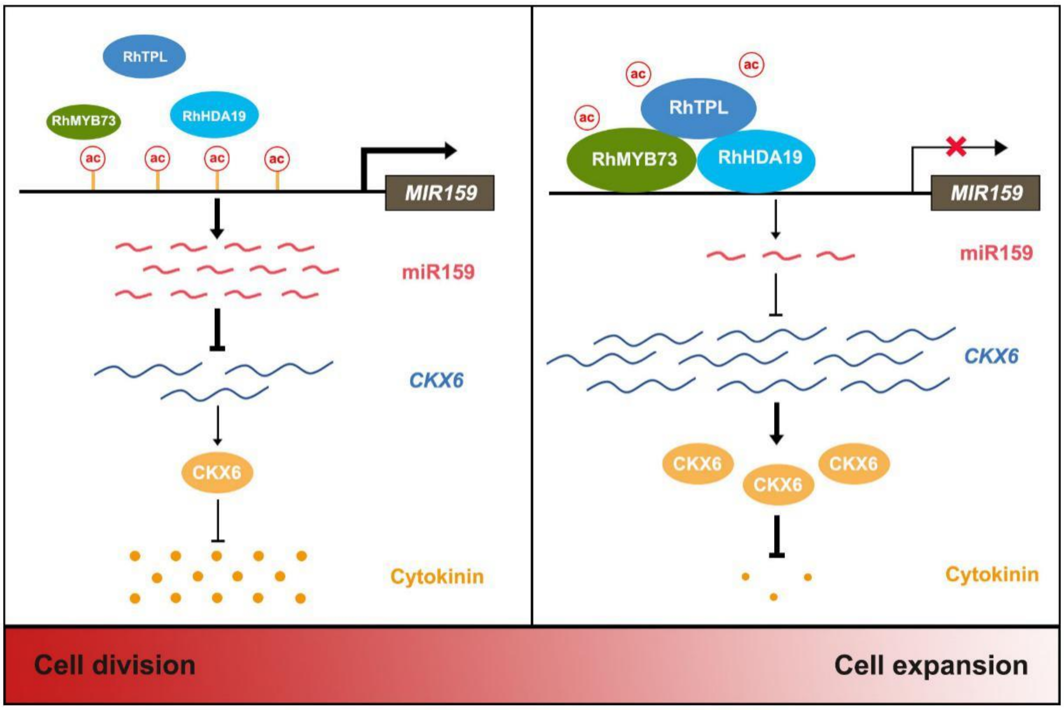


**Supplementary Figure 14 Proposed model of *MIR159-CKX6* module in governing onset of cell expansion *via* triggering rapid clearance of cytokinin in rose petals.**

Stage 0, low level of MYB73 cannot recruit TPL-HDA19 complex to the promoter of *MIR159*, high level of miR159 leads to cleavage of *CKX6* transcripts, and thus maintains high concentration of cytokinins, which promotes cell division. As petal development, increasing MYB73 recruits TPL-HDA19 complex to the promoter of *MIR159*, and thus suppresses expression of *MIR159*. Low level of miR159 releases accumulation of *CKX6* transcripts and thus initiates the clearance of cytokinins, which results in cease of cell division and startup of cell expansion.

# Uncropped Scans of All Blots and Gels in Supplementary Figures

**Supplementary Figure 8b**

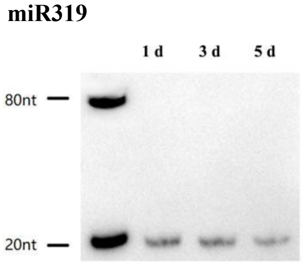
miR319
1 d
3 d
5 d
80nt
20nt

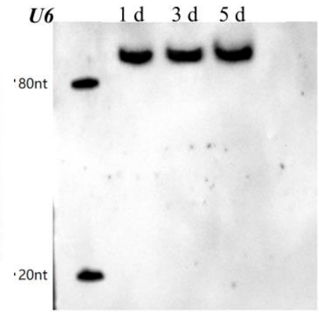
U6
1 d
3 d
5 d
80nt
20nt
