# Supplementary material for: Petal size is controlled by the MYB73/TPL/HDA19-miR159-CKX6 module regulating cytokinin catabolism in Rosa hybrida
Source: Nat Commun. 2023 Nov 4;14:7106. doi: 10.1038/s41467-023-42914-y (PMC10625627; doi:10.1038/s41467-023-42914-y)
Supplement: Supplementary file 3 — Description of Additional Supplementary Files [file 41467_2023_42914_MOESM3_ESM.pdf]

Description of Additional Supplementary Files

Supplementary Data 1. Differentially expressed genes (DEGs) between TRV and *STTM159* in the RNA-seq analysis.

Supplementary Data 2. Prediction analysis of miR159 target genes.

Supplementary Data 3. Cytokinin contents in petals of TRV and *STTM159* lines.

Supplementary Data 4. Proteins putatively binding to the promoter of *MIR159*.

Supplementary Data 5. RhMYB73-interacted proteins screening by IP-MS.

Supplementary Data 6. List of primers used.

Supplementary Data 7. List of genes used.
