# Supplementary material for: Petal size is controlled by the MYB73/TPL/HDA19-miR159-CKX6 module regulating cytokinin catabolism in Rosa hybrida
Source: Nat Commun. 2023 Nov 4;14:7106. doi: 10.1038/s41467-023-42914-y (PMC10625627; doi:10.1038/s41467-023-42914-y)
Supplement: Supplementary file 11 — Reporting Summary [file 41467_2023_42914_MOESM11_ESM.pdf]

nature portfolio

Corresponding author(s): Nan Ma and Xiaofeng Zhou

Last updated by author(s): Oct 22, 2023

Reporting Summary

Nature Portfolio wishes to improve the reproducibility of the work that we publish. This form provides structure for consistency and transparency in reporting. For further information on Nature Portfolio policies, see our Editorial Policies and the Editorial Policy Checklist.

Statistics

For all statistical analyses, confirm that the following items are present in the figure legend, table legend, main text, or Methods section.

n/a	Confirmed	
☐	☒	The exact sample size (*n*) for each experimental group/condition, given as a discrete number and unit of measurement
☐	☒	A statement on whether measurements were taken from distinct samples or whether the same sample was measured repeatedly
☐	☒	The statistical test(s) used AND whether they are one- or two-sided<br>*Only common tests should be described solely by name; describe more complex techniques in the Methods section.*
☒	☐	A description of all covariates tested
☐	☒	A description of any assumptions or corrections, such as tests of normality and adjustment for multiple comparisons
☐	☒	A full description of the statistical parameters including central tendency (e.g. means) or other basic estimates (e.g. regression coefficient) AND variation (e.g. standard deviation) or associated estimates of uncertainty (e.g. confidence intervals)
☐	☒	For null hypothesis testing, the test statistic (e.g. *F*, *t*, *r*) with confidence intervals, effect sizes, degrees of freedom and *P* value noted<br>*Give P values as exact values whenever suitable.*
☒	☐	For Bayesian analysis, information on the choice of priors and Markov chain Monte Carlo settings
☒	☐	For hierarchical and complex designs, identification of the appropriate level for tests and full reporting of outcomes
☒	☐	Estimates of effect sizes (e.g. Cohen's *d*, Pearson's *r*), indicating how they were calculated

Our web collection on statistics for biologists contains articles on many of the points above.

Software and code

Policy information about availability of computer code

Data collection

RNA-Seq data was acquired by Illumina NovaSeq 6000 (illumina, USA) sequencer;
Live luciferase images were collected by a CCD imaging machine (CHEMIPROHT 1300B/LND, 16 bit; Roper Scientific, Sarasota, FL, USA);
Relative activity of luciferase was measured by Glomax®-20/20 signal Tube Luminometer (Promega);
Microscopy images were collected by laser confocal fluorescence microscopy (Olympus, FV3000, Japan);
RT-qPCR data was collected by Step One PlusTM real-time PCR system (Applied Biosystems);
Cytokinins content was detected by MetWare (http://www.metware.cn/) based on the AB Sciex QTRAP 6500 LC-MS/MS platform (UPLC, ExionLC™ AD, https://sciex.com.cn/; MS, Applied Biosystems 6500; Triple Quadrupole, https://sciex.com.cn/);
Immunoblotting images were collected by Tanon-5200;
Petal size was measured by Image J software.

Data analysis

The secondary structure of rhy-MIR159 was analyzed by TBtools software version 1.0987663.
Prediction of miR159 target genes were used the psRNATarget website (https://www.zhaolab.org/ psRNATarget/);
Statistical analyses were used GraphPad Prism version 8.0; Two-sided Student's t-test and One-Way ANOVA with Tukey's multiple comparisons test were used for statistical analysis;
Graphs were generated by GraphPad Prism version 8.0;
Alignment was performed with ClustalW (https://www.genome.jp/tools-bin/clustalw);
Phylogenetic analysis was performed using MEGA software version X ;
Conserved domains were predicted by SMART analysis service (http://smart.embl-heidelberg.de/);
RNA-seq reads were aligned by a reference genome sequence (Rosa chinensis cv. Old Blush, GenBank ID 8255808) using the program Tophat v2.0.9; Novel transcripts are identified using Cufflinks version 2.1.1; Differential expression analysis was performed using the DESeq R package

(1.10.1); Gene Ontology (GO) enrichment analysis of the DEGs was implemented by the GOseq R packages based Wallenius non-central hyper-geometric distribution; KEGG (Kyoto Encyclopedia of Genes and Genomes) (http://www.genome.jp/kegg/) pathway enrichment analysis of DEGs was performed by using KOBAS software (2.0) and corrected P value cut-off was set at 0.05;
The MS/MS data was processed using MaxQuant search engine (v.1.5.2.8).

For manuscripts utilizing custom algorithms or software that are central to the research but not yet described in published literature, software must be made available to editors and reviewers. We strongly encourage code deposition in a community repository (e.g. GitHub). See the Nature Portfolio guidelines for submitting code & software for further information.

Data

Policy information about availability of data

All manuscripts must include a data availability statement. This statement should provide the following information, where applicable:

- Accession codes, unique identifiers, or web links for publicly available datasets
- A description of any restrictions on data availability
- For clinical datasets or third party data, please ensure that the statement adheres to our policy

RNA-seq data that support the findings of this study have been deposited in the NCBI Bioproject database under accession number PRJNA808873 (https://www.ncbi.nlm.nih.gov/bioproject?term=PRJNA808873). The raw mass spectrometry data of validation have been deposited to the ProteomeXchange Consortium under accession number PXD046197 (https://proteomecentral.proteomexchange.org/cgi/GetDataset?ID=PXD046197).

Human research participants

Policy information about studies involving human research participants and Sex and Gender in Research.

Reporting on sex and gender

Use the terms sex (biological attribute) and gender (shaped by social and cultural circumstances) carefully in order to avoid confusing both terms. Indicate if findings apply to only one sex or gender; describe whether sex and gender were considered in study design whether sex and/or gender was determined based on self-reporting or assigned and methods used. Provide in the source data disaggregated sex and gender data where this information has been collected, and consent has been obtained for sharing of individual-level data; provide overall numbers in this Reporting Summary. Please state if this information has not been collected. Report sex- and gender-based analyses where performed, justify reasons for lack of sex- and gender-based analysis.

Population characteristics

Describe the covariate-relevant population characteristics of the human research participants (e.g. age, genotypic information, past and current diagnosis and treatment categories). If you filled out the behavioural & social sciences study design questions and have nothing to add here, write "See above."

Recruitment

Describe how participants were recruited. Outline any potential self-selection bias or other biases that may be present and how these are likely to impact results.

Ethics oversight

Identify the organization(s) that approved the study protocol.

Note that full information on the approval of the study protocol must also be provided in the manuscript.

Field-specific reporting

Please select the one below that is the best fit for your research. If you are not sure, read the appropriate sections before making your selection.

☒ Life sciences ☐ Behavioural & social sciences ☐ Ecological, evolutionary & environmental sciences

For a reference copy of the document with all sections, see nature.com/documents/nr-reporting-summary-flat.pdf

Life sciences study design

All studies must disclose on these points even when the disclosure is negative.

Sample size	Sample size was selected based on our prior knowledge and experience on the experimental variability of experiments and the desire to get statistically significant data to support meaningful conclusions. This resulted sample sizes were from n=3 to n=10 to ensure the variation was captured. Sample sizes were described in Figure legends or in the main text.
Data exclusions	No data was excluded.
Replication	Each experiment was repeated at least two times and similar results were obtained.
Randomization	All plants were grown in the same standard conditions (please see the Methods section) and were placed randomly in the growth room. Comparison of phenotypes was based on the different genotypes (control vs gene-silenced lines).
Blinding	Data were collected according to the genotype of plants.

March 2021

Reporting for specific materials, systems and methods

We require information from authors about some types of materials, experimental systems and methods used in many studies. Here, indicate whether each material, system or method listed is relevant to your study. If you are not sure if a list item applies to your research, read the appropriate section before selecting a response.

Materials & experimental systems

n/a	Involved in the study
☐	☒ Antibodies
☒	☐ Eukaryotic cell lines
☒	☐ Palaeontology and archaeology
☒	☐ Animals and other organisms
☒	☐ Clinical data
☒	☐ Dual use research of concern

Methods

n/a	Involved in the study
☒	☐ ChIP-seq
☒	☐ Flow cytometry
☒	☐ MRI-based neuroimaging

Antibodies

Antibodies used

β-Tubulin (Abmart; catalog No. M20005; clone: monoclonal; dilution 1:2000)
FLAG (Abcam; catalog No. ab1162; clone: monoclonal; dilution 1:2,500);
GFP (Sigma-Aldrich; catalog No. G1544; clone: polyclonal; dilution 1:2000);
GFP-Trap® Agarose Beads (Chromotek; catalog No. gta; clone: polyclonal; dilution 1:200);
acetyl-Histone H3 (Lys9), H3K9ac (Abcam; catalog No. ab10812; clone: polyclonal; dilution 1:5,000);
acetyl-Histone H3 (Lys14), H3K14ac (Millipore; catalog No. 07-353; clone: polyclonal; dilution 1:5,000).

Validation

The β-Tubulin antibody was validated in Mikhaylova, M. et al. (2015) Resolving bundled microtubules using anti-tubulin nanobodies. Nat Commun 6, 7933.
The FLAG antibody was validated in Yan, A. et al. (2020) The atypical histone variant H3.15 promotes callus formation in Arabidopsis thaliana. Development 147:dev184895.
The GFP antibody was validated in Kim, S. et al. (2015) Nek2 activation of Kif24 ensures cilium disassembly during the cell cycle. Nat. commun. 6, 8087.
The GFP-Trap® Agarose Beads was validated in Leto, Dara E. et al. (2019) Genome-wide CRISPR Analysis Identifies Substrate-Specific Conjugation Modules in ER-Associated Degradation. Mol Cell. 73, 377.
The acetyl-Histone H3 (Lys9) antibody was validated in Zhuang, H. et al. (2020) NONSTOP GLUMES1 Encodes a C2H2 Zinc Finger Protein That Regulates Spikelet Development in Rice. Plant Cell. 32:392-413.
The acetyl-Histone H3 (Lys14) antibody was validated in Zhang, F. et al. (2016)EIN2-dependent regulation of acetylation of histone H3K14 and non-canonical histone H3K23 in ethylene signalling. Nat Commun. 7, 13018.
